# Clinical and radiological features of extra-pulmonary sarcoidosis: a pictorial essay

**DOI:** 10.1007/s13244-016-0495-4

**Published:** 2016-05-25

**Authors:** Stefano Palmucci, Sebastiano Emanuele Torrisi, Daniele Carmelo Caltabiano, Silvia Puglisi, Viviana Lentini, Emanuele Grassedonio, Virginia Vindigni, Ester Reggio, Riccardo Giuliano, Giuseppe Micali, Rosario Caltabiano, Cosma Andreula, Pietro Valerio Foti, Giovanni Carlo Ettorre, Simon LF Walsh, Carlo Vancheri

**Affiliations:** Radiodiagnostic and Radiotherapy Unit, University Hospital “Policlinico-Vittorio Emanuele”, Via Santa Sofia 78, 95123 Catania, Italy; Regional Centre for Interstitial and Rare Lung Diseases, Department of Clinical and Molecular Biomedicine, University Hospital Policlinico-Vittorio Emanuele, Via Santa Sofia 78, 95123 Catania, Italy; Unit of Diagnostic and Interventional Radiology, ARNAS Garibaldi, Catania, Italy; Section of Radiological Sciences, DIBIMEF, University Hospital “Paolo Giaccone” University of Palermo, Palermo, Italy; Department of G. F. Ingrassia, Section of Neurosciences, University Hospital Policlinico-Vittorio Emanuele, Via Santa Sofia 78, 95123 Catania, Italy; Dermatology Clinic, University of Catania, Catania, Italy; Department of G.F. Ingrassia, Institute of Pathology, University Hospital Policlinico-Vittorio Emanuele, Catania, Italy; Neuroradiology and Radiology, Anthea Hospital Bari, Gruppo Villa Maria, Puglia, Italy; Department of Radiology, Kings College Hospital Foundation Trust, Denmark Hill, London, UK

**Keywords:** Sarcoidosis, Granulomatous disease, chronic, Multidetector computed tomography, Magnetic resonance imaging, Positron-emission tomography

## Abstract

**Abstract:**

The aim of this manuscript is to describe radiological findings of extra-pulmonary sarcoidosis. Sarcoidosis is an immune-mediated systemic disease of unknown origin, characterized by non-caseating epitheliod granulomas. Ninety percent of patients show granulomas located in the lungs or in the related lymph nodes. However, lesions can affect any organ. Typical imaging features of liver and spleen sarcoidosis include visceromegaly, with multiple nodules hypodense on CT images and hypointense on T2-weighted MRI acquisitions. Main clinical and radiological manifestations of renal sarcoidosis are nephrolithiasis, nephrocalcinosis, and acute interstitial nephritis. Brain sarcoidosis shows multiple or solitary parenchymal nodules on MRI that enhance with a ring-like appearance after gadolinium. In spinal cord localization, MRI demonstrates enlargement and hyperintensity of spinal cord, with hypointense lesions on T2-weighted images. Skeletal involvement is mostly located in small bone, showing many lytic lesions; less frequently, bone lesions have a sclerotic appearance. Ocular involvement includes uveitis, conjunctivitis, optical nerve disease, chorioretinis. Erythema nodosum and lupus pernio represent the most common cutaneous manifestations encountered. Sarcoidosis in various organs can be very insidious for radiologists, showing different imaging features, often non-specific. Awareness of these imaging features helps radiologists to obtain the correct diagnosis.

***Teaching Points*:**

• *Systemic sarcoidosis can exhibit abdominal, neural, skeletal, ocular, and cutaneous manifestations.*

• *T2 signal intensity of hepatosplenic nodules may reflect the disease activity.*

• *Heerfordt’s syndrome includes facial nerve palsy, fever, parotid swelling, and uveitis.*

*• In the vertebrae, osteolytic and/or diffuse sclerotic lesions can be found.*

• *Erythema nodosum and lupus pernio represent the most common cutaneous manifestations*.

## Introduction

Sarcoidosis is a multi-systemic disorder of unknown cause, pathologically characterized by the accumulation of inflammatory cells forming non-caseating granulomas. Lesions can be located in any organ but in about 90 % of patients, granulomas affect the lungs or the related lymph nodes [1].

According to Geize et al., sarcoidosis can be encountered in extra-pulmonary locations, in approximately 30 % of cases [2]. Moreover, the study entitled “A Case Control Etiologic Study of Sarcoidosis” (ACCESS) provided an accurate analysis regarding distribution of the disease: in 736 sarcoidosis cases, 699 patients showed thoracic disease, and 368 out of the 736 patients had concomitant extra-thoracic disease [3–5]; isolated extra-pulmonary disease was found only in a small percentage of cases (2 %) [3, 5].

The systemic location of the disease can be very insidious for radiologists: a wide variety of imaging features are found, often non-specific, which can mimic other pathological conditions [6, 7]. Therefore, the aim of this pictorial essay is to describe the most important clinical features and the main radiological findings of sarcoidosis in various organs, in order to help radiologists in the identification of the disease.

## Epidemiology

The disease affects young men and women aged between 25 and 40 years, without major differences in ethnicity. Some studies do show a greater incidence in females [8]. In about 30 % of cases, there is a second peak incidence from 50 to 65 years of age. This second peak mostly affects women showing clinical characteristics that differ from those of younger patients [9, 10]. The annual incidence varies widely from country to country. In northern Europe it is about 5-40/100,000, which represents the highest value reported; in Japan the incidence is lower, with a value of about 1-2/100,000. Afro-Americans are the most affected (35.5/100,000), whereas white Americans have an incidence of 10.9/100,000 [8].

Normally, sarcoidosis shows a variable clinical course; it may also exhibit different extra-thoracic manifestations. These vary widely according to ethnicity: for example, involvement of the eye, liver, bone marrow, extra-thoracic lymph nodes, and skin has been more statistically associated with African Americans than with Caucasians [3, 5].

Cardiac and ocular diseases are frequently encountered in Japanese patients, erythema nodosum in northern Europeans, and ocular and granulomatous skin involvement in black patients [11–13]. In addition, regarding cutaneous lesions, Lupus pernio has been frequently observed in Puerto Ricans.

Extra-thoracic sarcoidosis involves females more frequently than males [3].

## Extra-pulmonary sarcoidosis: clinical and imaging features

Extra-pulmonary sarcoidosis has been found in 30 % of patients with the disease [2]. The most common site reported in the paper by Gezer et al. is the abdomen, which includes liver, spleen, biliary tree, peritoneum, and lymphatic sarcoidosis [2].

The cutaneous system and the eyes are involved with a frequency of 25 % [14, 15]. Following data reported in the paper by Rao et al., the third most affected organ is the eye, with a frequency of involvement ranging from 10 to 60 % [11]. However, according to data reported by Koyama et al., ocular sarcoidosis is found with higher frequency, being about 80 % [16]. Skin lesions are found in 20 to 35 % of patients with sarcoidosis [11, 17, 18]; similarly, a frequency of 25 % for cutaneous involvement has been reported in other papers [16, 19].

The following sections below describe more in detail clinical and radiological features of systemic sarcoidosis (Table 1), focusing on abdominal, neural, musculo-skeletal, cutaneous, ocular, and cardiac manifestations.

**Table 1:** Main clinical and radiological features of sarcoidosis in different organs

Location	Clinical features	Imaging findings
Liver	Hepatomegalia, enlargement of abdominal lymph nodes	Round or oval shaped hypoechoic nodules at ultrasonography; Hypodense nodules on CT images; Hypointense nodules on T2-weighted MRI sequences and on gadolinium-enhanced T1-weighted MRI acquisitions
Spleen	Patients can be asymptomatic or complain of fever, weight loss, and malaise	Ultrasound reveals splenomegaly with small hypoechoic nodules; Hypodense nodules after contrast-enhanced CT images; Nodular lesions with low signal in all sequences and visible in the early gadolinium-enhanced T1 images
Gastro-intestinal Tract	Disease involves gastric antrum, biliary tree, and parotid glands	Small ulcerations of gastrointestinal tract; Enlargement of parotid glands can be observed, with inhomogeneous or nodular pattern on enhanced CT images; Increased T2-signal intensity on MRI acquisitions of parotid glands
Lymphatic System	Lymphadenopathy or increased number of normal sized nodes	Enlarged nodes can be located in the periportal or para-aortocaval region, close to the liver hilus, adjacent to the celiac trunk or pancreas
Peritoneum	Ascites and multiple nodules	Hypoattenuating nodules on CT images, fluid accumulation in the abdomen
Kidneys	Nephrolithiasis, nephrocalcinosis, nephrogenic diabetes insipidus, renal insufficiency, acute interstitial nephritis	“Striated nephrogram” can be found on CT and MRI acquisitions; Granulomatous pseudotumor appears as hypo-/iso-/hyperatteanuating area on unenhanced CT scans, hypodense after contrast administration; Granulomatous pseudotumor shows low signal on early and delayed images after gadolinium administration
Central Nervous System	Signs of cranial nerve involvement; headache, seizure, meningeal irritation	Lesions are hyperintense on T2-weighted MRI images, located in the white and grey matter; Leptomeningeal localizations are more visible after contrast injection, showing increased signal on enhanced T1 acquisitions
Bone	Hands and feet are the most common locations	On conventional radiography, lesions produce a lacy pattern of osteolytic areas in the digits; Large bone and axial skeleton lesions can be detected as radiolucent or sclerotic areas
Heart	Conduction disturbances and arrhythmias, pericarditis	Granulomatous lesions are observed as areas of focal enhancement on cardiac MRI, most frequently located in myocardial wall or subepicardial region
Skeletal Sarcoidosis	Involves hand and feet; large bones and axial skeleton involvement is uncommon	Sclerotic areas consisting of hyperdense homogeneous areas, round or oval in shape; osteolysis produces a hypodense appearance Lesions with high signal intensity on T2-weighted images, high-density proton sequences and STIR acquisitions; on T1-weighted images, lesions are generally hypointense

## Abdominal sarcoidosis

### Hepatic sarcoidosis

Hepatic sarcoidosis occurs with a prevalence of 1-40/100,000; it generally involves young people [20]. The disease is underestimated: indeed, involvement of hepatic parenchyma is encountered—in autopsy specimens—in 50–80 % of cases [16]. In 2–60 % of patients, laboratory tests show abnormal levels, indicating an organ dysfunction [21]. Portal hypertension, cirrhosis and chronic cholestatic disease are rarely reported from patients with hepatic sarcoidosis [22].

Commonly, imaging demonstrates hepatomegaly, with homogeneous appearance of parenchyma [16]; very often, this radiological feature is associated with splenomegaly and enlargement of abdominal lymph nodes [20], which are encountered close to the liver hilus or in celiacal region. However, in 5–15 % of cases, multiple nodular granulomas, ranging from 1 to 2 mm to several centimeters in diameter, become visible on CT and MRI [16]. Nodular involvement—according to Karagiannidis et al.—can be detected in low percentage (<5 % of cases) [20].

**Fig. 1:**
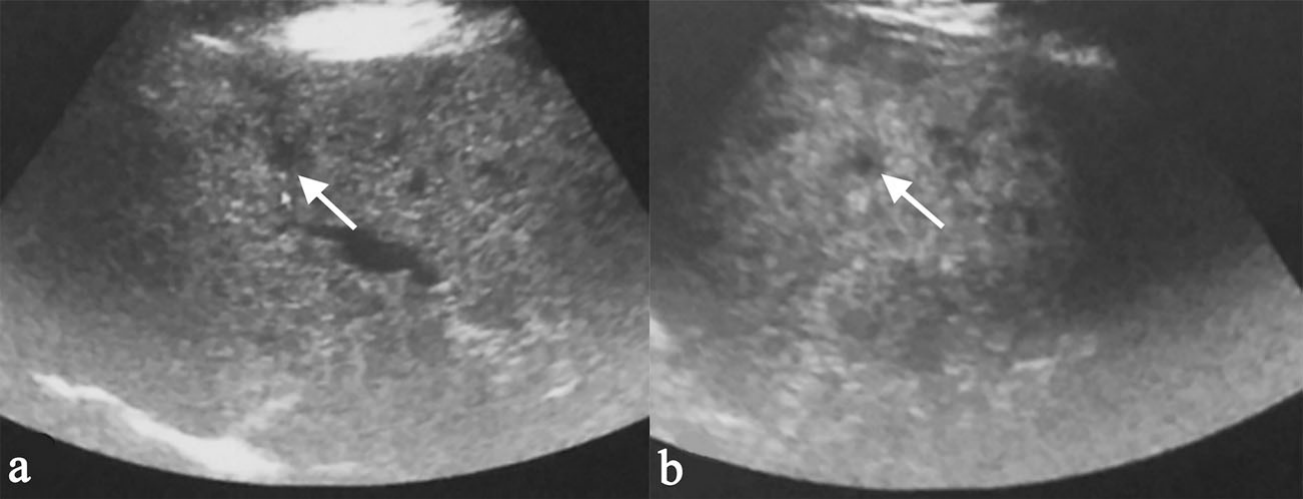
A 61-year-old female patient affected by pulmonary sarcoidosis, proved by biopsy. Routine abdominal ultrasound exam (on Fig. 1a and b) shows multiple, small, randomly distributed, hypoechoic nodules in the liver parenchyma. Nodules were histologically related to sarcoidosis

**Fig. 2:**
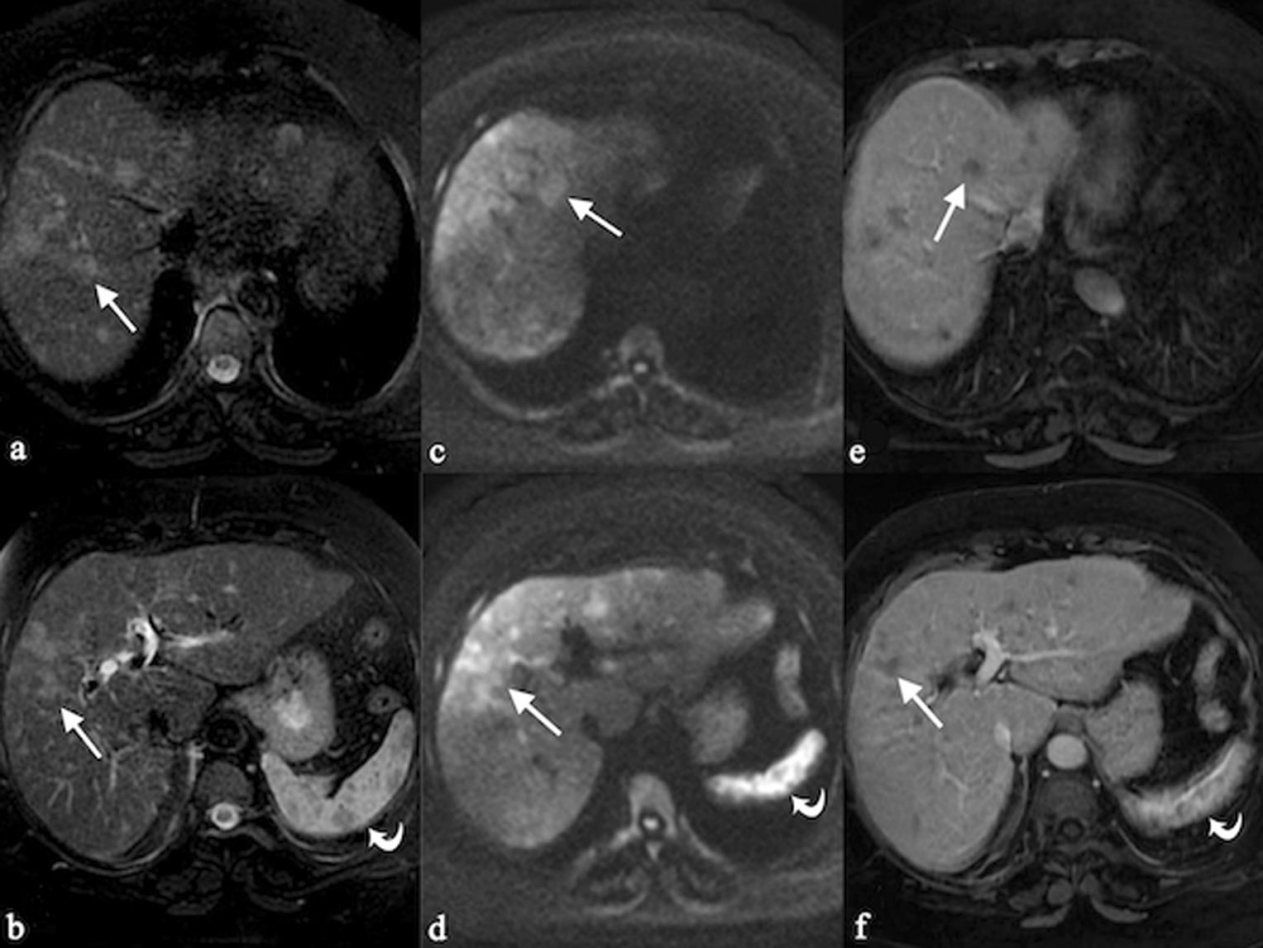
Hepatosplenic sarcoidosis. Liver MRI demonstrates multiple, scattered, small, hyperintense nodules (*white arrows*) on T2-weighted images (Fig. 2a and b) and on diffusion weighted imaging (DWI) (Fig. 2c and d); after gadolinium administration, nodules appear hypointense (Fig. 2e and f). In hepato-splenic sarcoidosis, T2 signal intensity reflects the degree of activity of disease: nodules can appear hyperintense in case of inflammation due to oedema and high vascular permeability; they have hypointense signal, when fibrosis is prevalent (see *curved arrows* for nodular lesions of the spleen)

Granulomatous lesions are located in the portal and periportal spaces of hepatic sinuses and generally exhibit “an identical stage of maturation” [20, 23]. They may be seen as round or oval shaped hypoechoic nodules at ultrasound (Fig. 1), involving hepatic parenchyma in a diffuse or limited form. These nodules are hypodense on CT images and show a low signal on MRI scan, especially in T2-weighted fat-saturated acquisitions. The nodules are generally hypointense on gadolinium-enhanced T1-weighted acquisitions [23–25]. However, signal intensity depicted on T2-weighted images reflects the degree of activity of disease: nodules can appear hyperintense in case of inflammation, due to oedema and high vascular permeability (Fig. 2) [26]. Similarly, on diffusion weighted imaging (DWI) acquisition, nodules with inflammation appear as high signal intensity lesions, with restriction on apparent diffusion coefficient (ADC) map. Fibrotic nodules show low signal on T2-weighted and diffusion sequences [26].

### Splenic sarcoidosis

Splenic sarcoidosis is generally associated with lung disease; however, normal chest radiography is observed in one quarter to one third of patients with splenic sarcoidosis [27]. Splenic involvement has been reported in a variable percentage, ranging from 24 to 59 % of biopsies [28–30]; at autopsy, the frequency of sarcoid lesions in the splenic parenchyma is about 41 % [31].

Patients can be asymptomatic, or complain of fever, weight loss, and malaise [21, 32]. In the series reported by Warshauer et al., splenomegaly has been found in one third of cases [32]. Hepatic and splenic contemporary involvement has been reported in 5–15 % of cases [32].

**Fig. 3:**
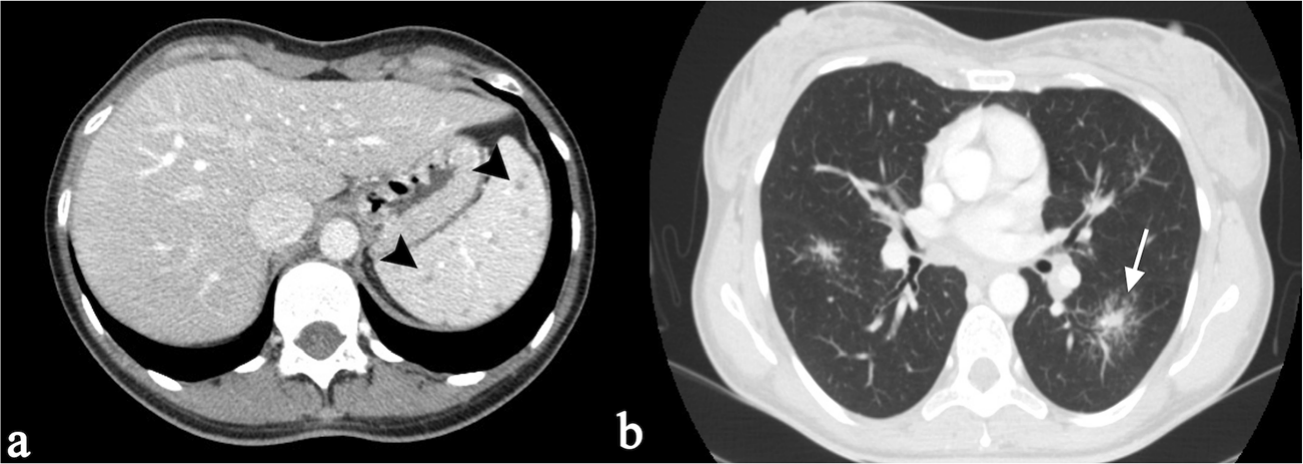
A 40-year-old female patient affected by pulmonary and splenic sarcoidosis. Figure 3a shows splenic sarcoidosis in a nodular pattern; the nodules (*black arrowheads*) are revealed as small hypodense nodules on contrast-enhanced CT images. Ill-defined pulmonary opacities, with small nodules around, are also depicted on Fig. 3b, resembling the typical sarcoid galaxy sign (*white arrow*)

**Fig. 4:**
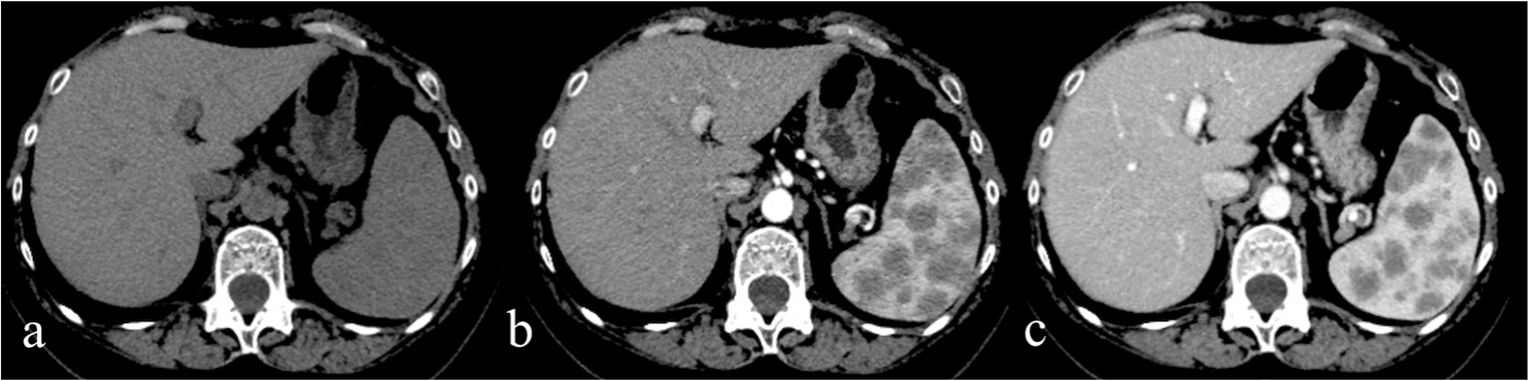
Splenic sarcoidosis in a 58-year-old female. Splenic parenchyma, on Fig. 4a, appears slightly inhomogeneous. After contrast administration (Fig. 4b and c), large nodules (exceeding 1 cm) with hypodense appearance are clearly depicted

Splenic sarcoidosis can be present in a homogeneous fashion or in a nodular pattern [2, 27, 33]. At ultrasound, splenic lesions may be detected as small nodules, with hypoechoic attenuation in comparison to surrounding parenchyma. On CT images, lesions are generally revealed as small hypodense nodules after contrast administration (Fig. 3); nodules can be larger than 1 cm in size, with a tendency to confluence (Fig. 4).

**Fig. 5:**
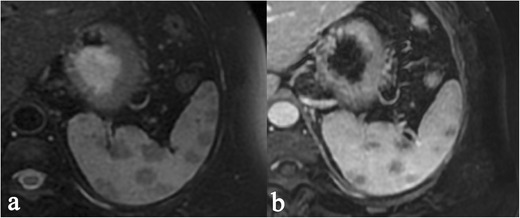
MRI appearance of a splenic sarcoidosis (same patient as Fig. 2). The visualization of the nodules reaches an optimal level on T2-weighted acquisition with fat suppression (Fig. 5a) and in the early gadolinium-enhanced T1 images (Fig. 5b)

On MRI acquisitions, nodular lesions show low signal in all sequences [34]; the visualization reaches an optimal level on T2-weighted acquisition with fat suppression and in the early gadolinium-enhanced T1 images (Fig. 5) [34, 35]. It has recently pointed out that MRI is able to monitor the activity of the disease. On T2-weighted images, nodules can appear hyperintense in cases of inflammation due to oedema and high vascular permeability; also, on DWI acquisitions, inflammation of nodules show high signal intensity, with restriction on ADC map [26].

**Fig. 6:**
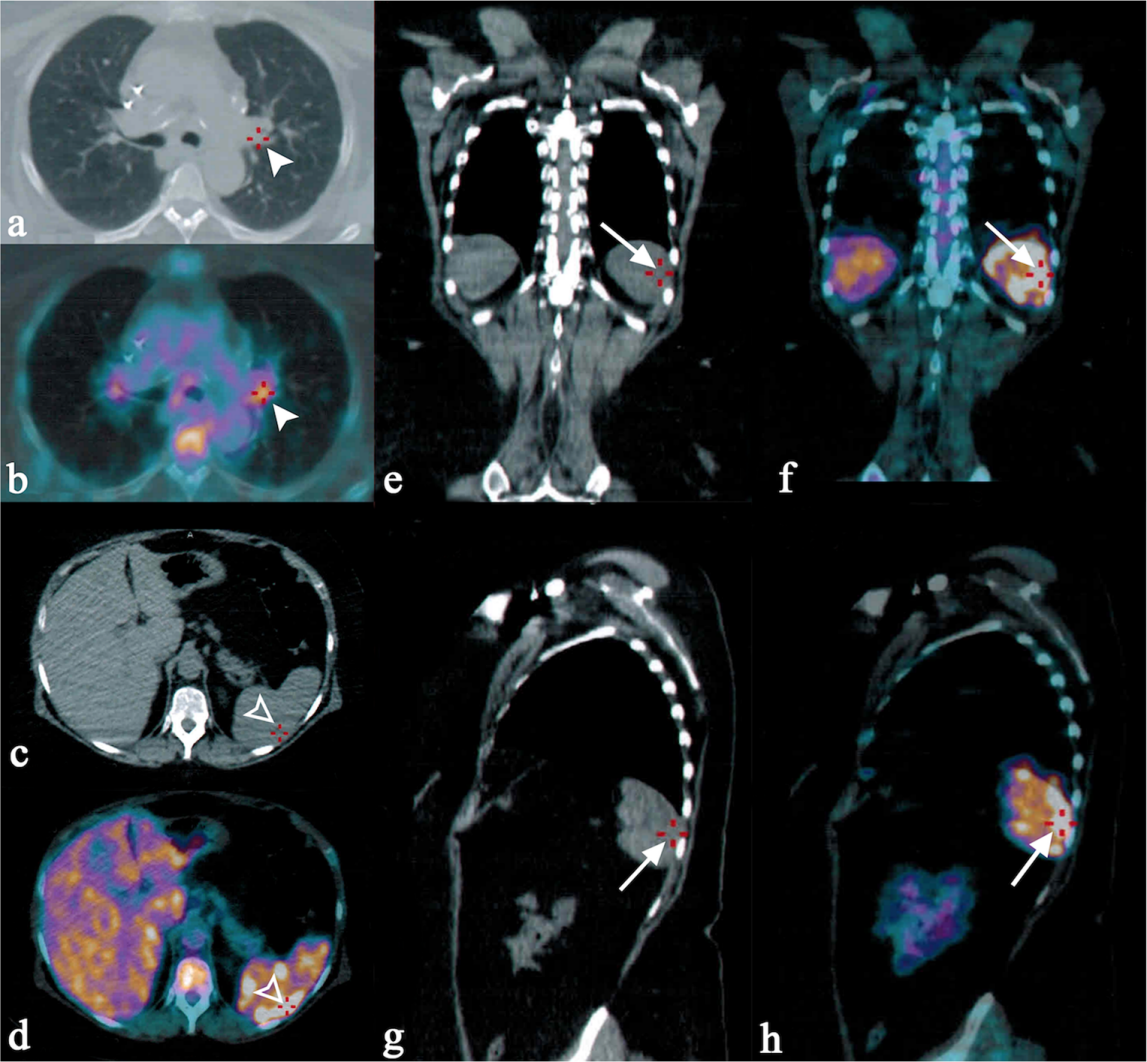
FDG uptake on PET-CT in a 57-year-old woman affected by pulmonary and splenic sarcoidosis. Increased metabolic activity is appreciable in the left pulmonary hilum, due to pathological lymph nodes (*white arrowheads* on Fig. 6a–b). Similarly, splenic foci of increased metabolic activity are clearly seen on axial CT-PET acquisitions (empty *white arrowheads* respectively on Fig. 6c–d), and on coronal and sagittal reformatted images (*white arrows* in Fig. 6e–h)

Parenchymal lesions show increased FDG uptake on PET-CT [36]: low-density areas on CT reveals multiple foci of increased metabolic activity on PET images (Fig. 6).

### Gastrointestinal sarcoidosis

Among abdominal sarcoidosis, autopsy revealed only a small percentage of cases (5 %) located in pancreatic tissue, intestinal tract, and testes [21]. The gastric antrum is the area most frequently involved [11]. Along the gastrointestinal tract, small ulcerations and mucosal thickening can be observed.

The biliary tree may be involved in extra-hepatic or intra-hepatic pattern. Granulomatous lesions develop in the portal triad, causing a cholestatic pattern.

Extra-hepatic involvement of the biliary tree can be caused by multiple small granuloma of the wall; the radiological appearance in these cases may simulate a cholangiocarcinoma; in many cases, the biliary tree may be compressed by enlarged abdominal nodes.

**Fig. 7:**
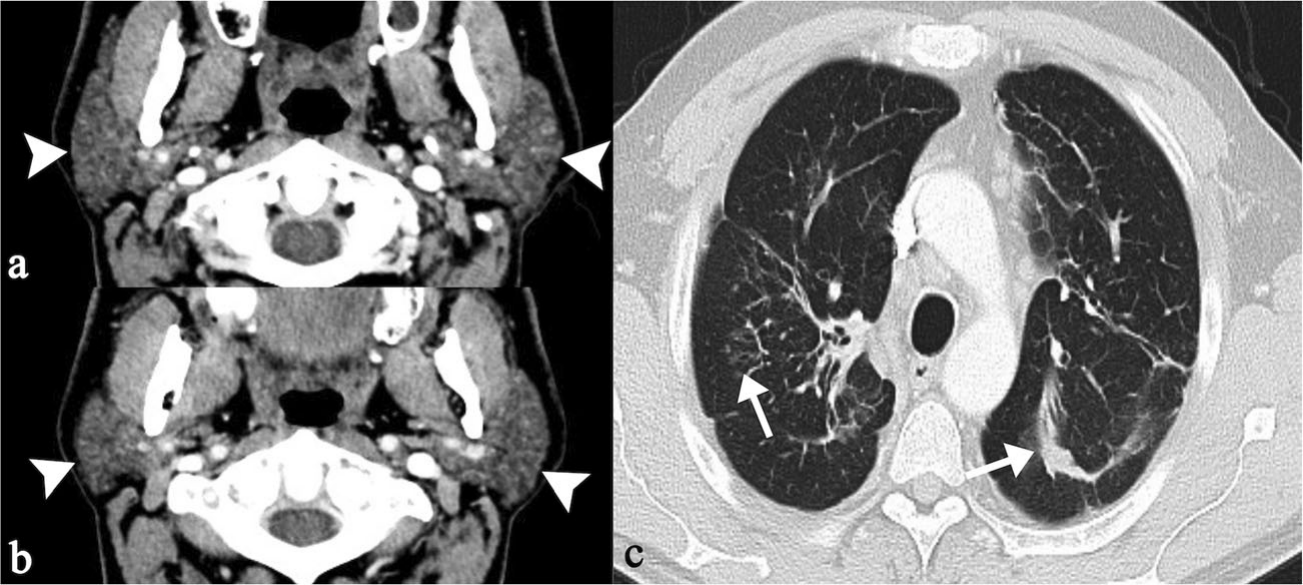
Parotid gland sarcoidosis (histologically proven). On enhanced-CT images, parotid glands show inhomogeneous pattern (*white arrowheads*), with multiple and punctuate small hypodense lesions. Figure 7c show typical pattern of thoracic disease on CT acquisitions

**Fig. 8:**
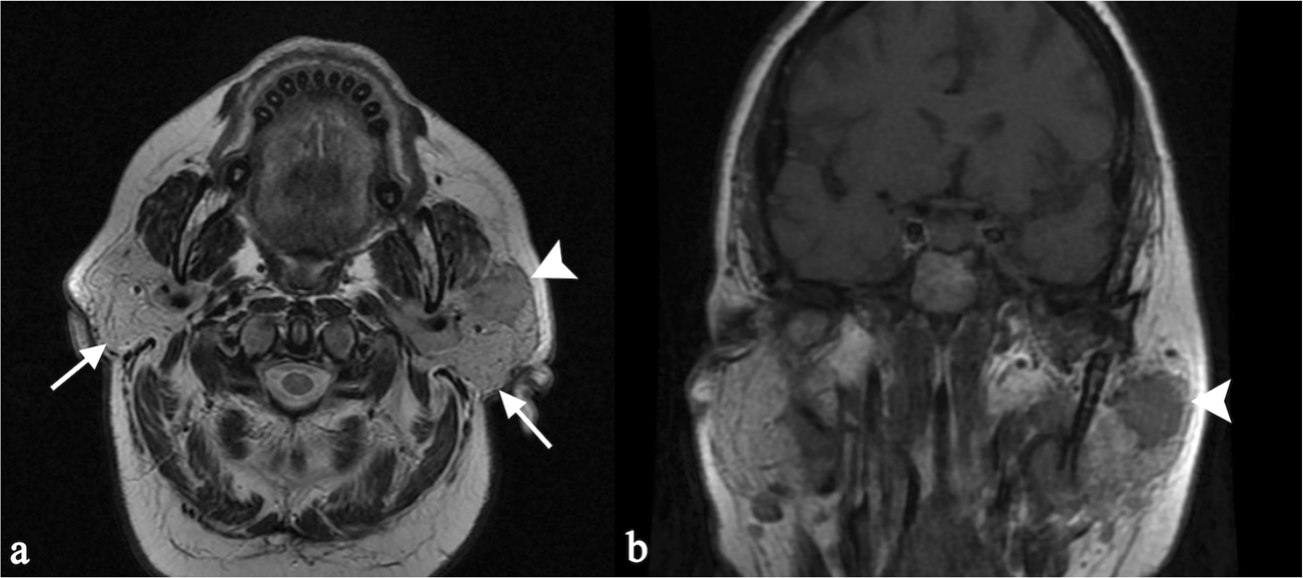
Parotid sarcoidosis (histologically proven), with enlargement of glands (*white arrows*). Nodular lesion is clearly depicted in the parenchyma of left parotid (*white arrowheads* in Fig. 8a and b)

According to Koyama et al., in 6 % of cases parotid glands may have sarcoidosis involvement; generally, the disease is found bilaterally, with enlargement of glands [16]. An inhomogeneous pattern can be observed on enhanced CT images (Fig. 7); increased T2-signal intensity and nodular lesions are observed on MRI acquisitions (Fig. 8). Increased uptake of Gallium-67 reproduces the typical “Panda” sign, even if this radionuclide accumulation is also described in other pathological entities (lymphoma and HIV infection) [36].

### Lymphatic and peritoneal sarcoidosis

Lymphatic abnormalities have been already described as abdominal manifestations of sarcoidosis, consisting of “lymphadenopathy or increased number of normal sized nodes” [37]; lymph nodes are generally 1–2 cm in size. In a previous experience, abdominal adenopathy was reported in approximately 30 % of patients; in these case series, adenopathy was defined as “two or more nodes with a short axis diameter greater than 6 mm” [21].

**Fig. 9:**
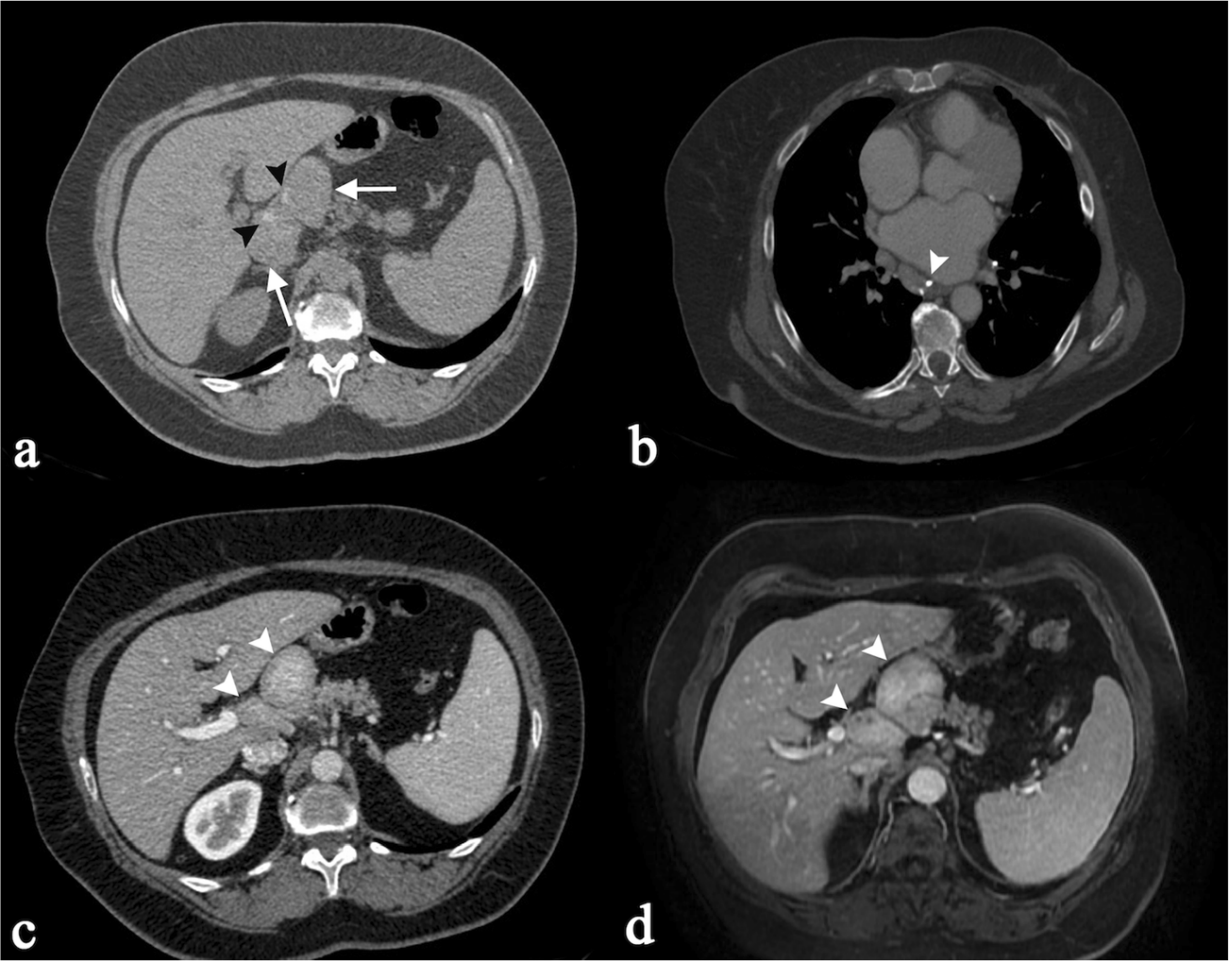
A 66-year-old female patient affected by sarcoidosis, which involves mediastinal and abdominal lymph nodes (histologically proven). Unenhanced abdominal CT image (Fig. 9a) demonstrate enlarged lymph nodes (*white arrows*), located in perihepatic region and close to the liver hilus; inside these lymph nodes, very small calcifications are recognizable (*black arrowheads*). Chest CT-scan shows mediastinal lymphadenopathy, with some punctate calcifications (*white arrowhead* on Fig. 9b). After contrast administration, both CT and MRI acquisitions show inhomogeneous enhancement of enlarged lymph nodes (*white arrowheads* on Fig. 9c and d)

**Fig. 10:**
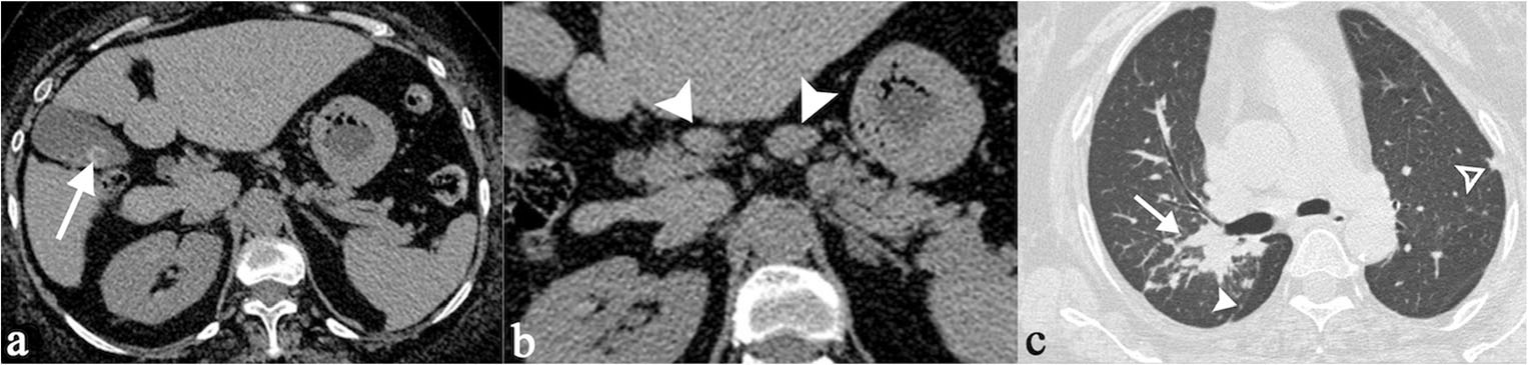
Incidental sarcoidosis diagnosis in a 59-year-old woman complaining of acute abdominal pain. Unenhanced CT scan shows a gallbladder calculus (*white arrow* on Fig. 10a) and some peri-pancreatic lymph nodes (*white arrowheads* on Fig. 10b); then CT scan extended through the chest (Fig. 10c) showed multiple, small subpleurical nodules. Histological exam confirmed diagnosis of sarcoidosis for the mentioned peri-pancreatic lymph nodes

**Fig. 11:**
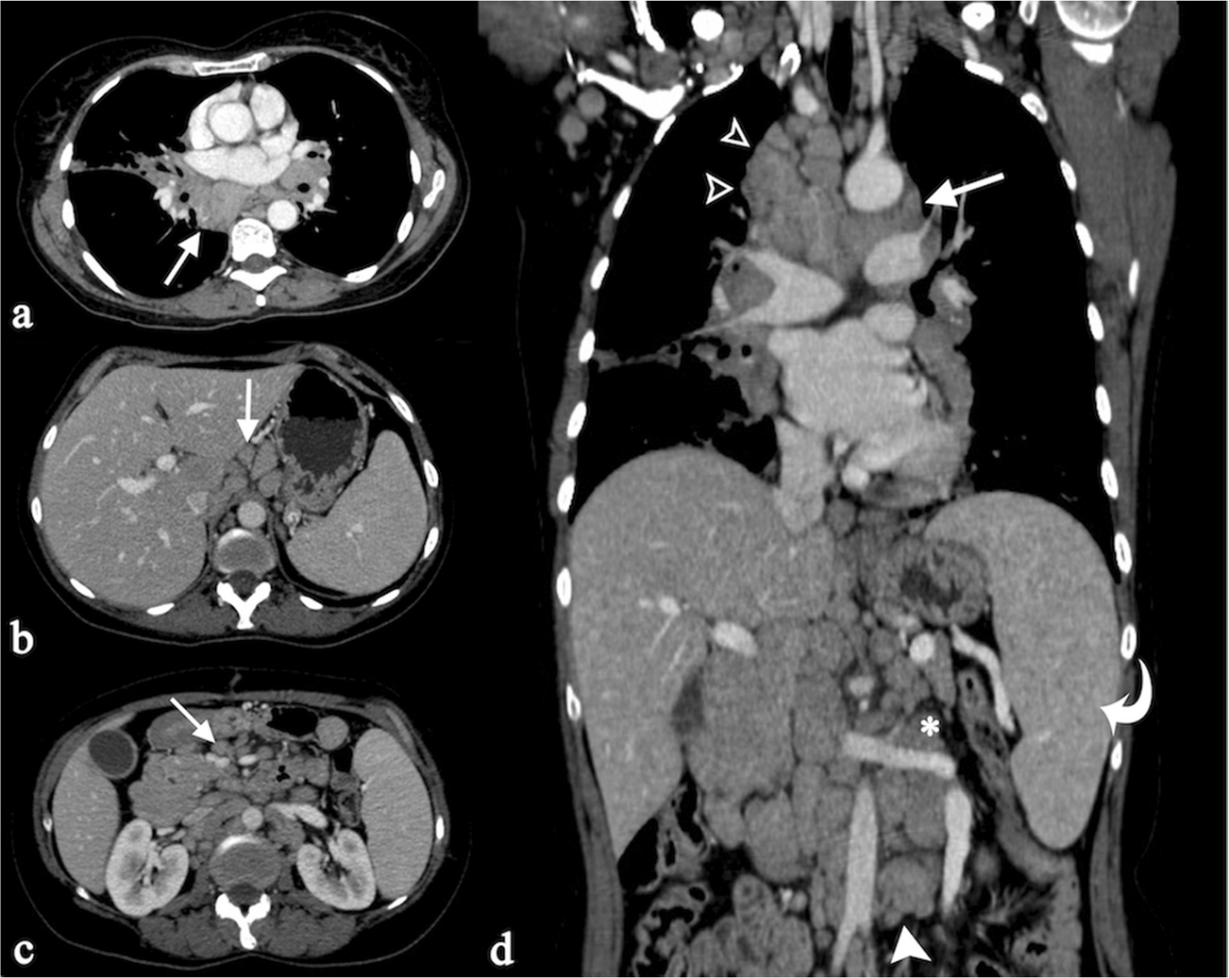
CT scan of a 55-year-old woman with multiple histologically proven sarcoidosis lymphadenopathy. Contrast-enhanced axial CT images (Fig. 11a–c) show multiple enlarged and confluent lymph nodes, located in the mediastinum (Fig. 11a), in the celiac region (Fig. 11b), and in the mesentery (Fig. 11c). Coronal MPR image (Fig. 11d) clearly depicts lymphadenopathy in the right side of the mediastinum, in the aorto-pulmonary recess (*white arrow*), at the right paratracheal level (*empty* *white arrowheads*), and in the left perirenal space (*white asterisk*). Spleen enlargement can also be detected (*white curved arrow*)

Enlarged nodes can be observed in different locations: in the periportal region, close to the liver hilus (Fig. 9), or between portal vein and vena cava. They can be found adjacent to the celiac trunk or pancreas (Fig. 10), or in a paraaortic disposition. Lymph nodes are also found around iliac vessels, or in the mesentery. A diffuse abdominal nodes involvement can be observed (Fig. 11). Necrosis or calcifications are rare in sarcoidosis [21].

Lymphatic involvement is frequently associated with pulmonary disease, and this can be very helpful for radiologists in order to achieve a correct diagnosis. When the disease is limited to the lymphatic system in mediastinum and in the abdomen, a differential diagnosis from other disorders, e.g. lymphoma, may be very difficult.

Peritoneal sarcoidosis is extremely rare [2, 38–41]. It can appear with ascites and multiple nodules: these imaging features—as reported by Gezer et al.—should be differentiated from other pathological conditions, which include carcinomatosis, tuberculosis, and fungal infections [2].

### Renal sarcoidosis

In sarcoidosis, the real incidence and prevalence of renal involvement is difficult to establish, because the disease can occur in a variable manner; in most cases, it has been found in 35 to 50 % of patients [42]. Main clinical manifestations are nephrolithiasis, nephrocalcinosis, nephrogenic diabetes insipidus, renal insufficiency, and acute interstitial nephritis with or without granuloma; however, many patients with renal involvement are asymptomatic.

The increased levels of 1,25-dihydroxyvitamin, produced by mononuclear cells trapped in pulmonary alveoli, determines a greater absorption of calcium: this leads to hypercalcemia and consequential nephrolithiasis and nephrocalcinosis [43].

The prevalence of interstitial nephritis in sarcoidosis oscillates from 7 to 27 % [44, 45]. The renal involvement may be with or without granuloma. Granuloma interstitial nephritis is the typical histological finding and is defined “naked with no cuff of inflammatory cells with presence of asteroid bodies and calcification” [46]. This lesion differs form tuberculosis granuloma for the absence of necrosis.

Enhanced CT images may show signs of interstitial nephritis [21, 47, 48] with the typical “striated nephrogram” represented by ill-defined hypodense lines into the renal parenchyma. Diffusion MRI sequences show interstitial nephritis as hyperintense areas, with signal restriction on apparent diffusion coefficient map.

**Fig. 12:**
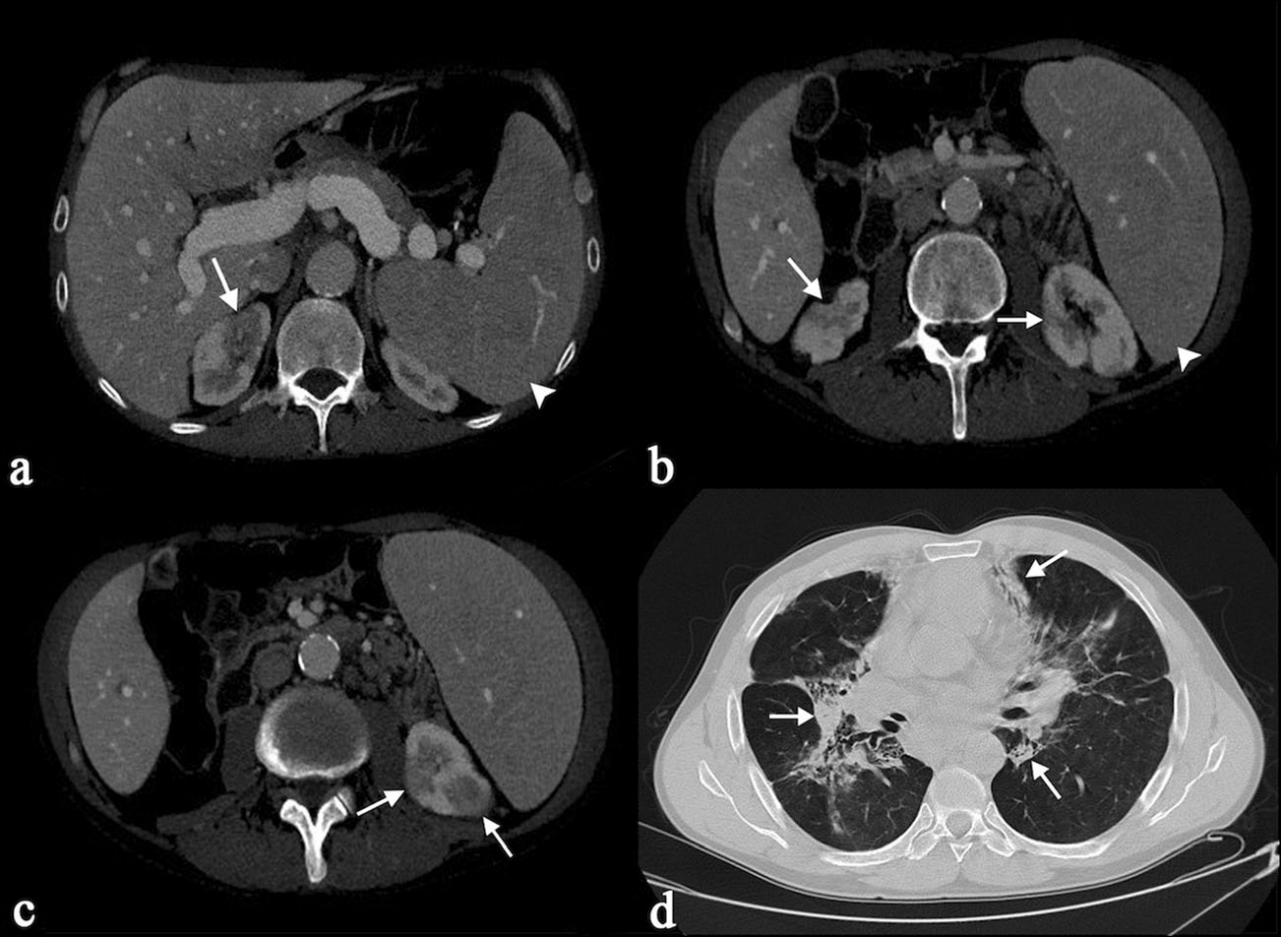
Multiorgan sarcoidosis, with multiple hypodense lesions in the renal parenchyma (*white arrows* in Fig. 12a–c). In the left kidney, in the lower part of the parenchyma, lesions have nodular appearance. Splenomegaly can also be observed (*white arrowheads*). In the Fig. 12d, a sarcoidosis pattern is observed on HRCT images (*white arrows*). Sarcoid lesions in the renal parenchyma were confirmed by biopsy

Rarely, radiological features of renal sarcoidosis can be represented by granulomatous pseudotumour; these lesions are generally incidentally discovered during CT examination. They can appear as hypo-, iso-, or hypeattanuating areas on unenhanced CT scans; after contrast administration, they are hypodense to the surrounding renal parenchyma (Fig. 12) [48]. Differential diagnosis includes lymphoma or other tumours. On MRI acquisition, lesions show heterogeneous signal on unenhanced sequences and appear hypointense on early and delayed images acquired after gadolinium administration [48].

The urinary manifestations of sarcoid nephritis are nonspecific: the alterations of the urine analysis are similar to those of chronic tubular pathologies. Biopsy is the only way to make a diagnosis, showing normal glomeruli with interstitial infiltration of mononuclear cells and non-caseating granulomas; occasionally, it may be also depicted with glomerular disease [49, 50].

## Neurosarcoidosis

Central nervous system (CNS) involvement is reported in 25 % of cases at autopsy; in a post-mortem series, neural sarcoidosis was detected with a frequency of 14–27 % [51–54]. Symptoms due to neurosarcoidosis are not frequently observed, being encountered in less than 10 % of patients [16].

**Fig. 13:**
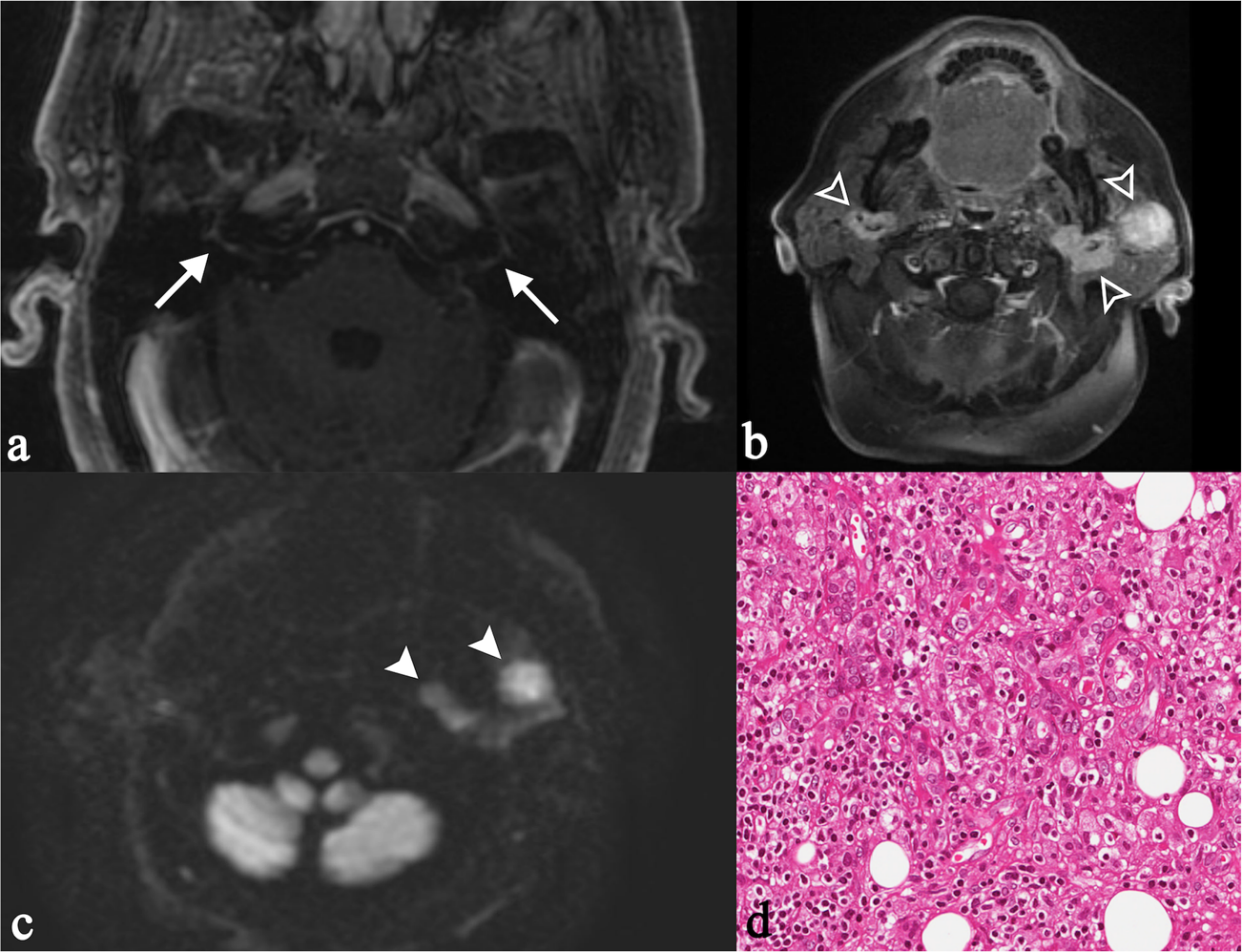
“Heerfordt’s syndrome” in a 60-year-old woman. Clinically, the patient complained of sudden appearance of blurred vision, headache, and mouth deviation to the left after fever. MRI shows bilateral enhancement of facial nerve (*white arrows* on Fig. 13a), recognizable on T1-weighted image obtained after gadolinium administration. She showed a complete facial palsy and a bilateral enlargement of the cheeks. Physical examination revealed an unpainful enlargement of the left parotid gland, left facial nerve and left abducent nerve palsy. Nodular lesions were found in the parotid glands (same patient in Fig. 8), with evident contrast enhancement on T1-weighted acquisitions after contrast enhancement (Fig. 13b, empty *white arrowhead*) and on DWI sequences (Fig. 13c, *white arrowheads*). Chest CT scan and fibrobronchoscopy with bronchoalveolar lavage were suggestive of sarcoidosis. The biopsy of the left parotid gland depicted dense, non-caseating granulomatous infiltrate (Fig. 13d). Lymphocytes and epithelioid histiocytes with abundant eosinophilic cytoplasm and oval vesicular nuclei are observed (H&E 250X). The clinical, radiological and histopathological patterns were consistent with the diagnosis of Heerfordt’s syndrome

The disease shows a special predilection for the basal cisterna of the brain; cranial nerve involvement is frequent, influencing the clinical manifestation of disease. Facial nerve palsy, for example, is a typical manifestation of brain sarcoidosis in young adults [16]. It may be associated with chronic fever, swelling of parotid gland and uveitis, configuring the typical “Heerfordt’s syndrome” (Fig. 13).

Other symptoms associated with neural sarcoidosis are headache, seizure, and signs of meningeal irritation [55, 56]. Involvement of optical nerve leads to loss of vision, with a poor prognosis [57].

Diagnosis of sarcoidosis can be performed following the criteria reported in the study by Pawate et al., in which the presence of neurosarcoidosis is evaluated as definite, probable, and possible. According to their criteria [57], the disease is graded as “definite” when detected on histological specimens. It is labelled as “probable” when: a) laboratory tests are positive for CNS inflammation (elevated levels of cerebrospinal fluid protein or MRI features compatible with neurosarcoidosis) b) detection of systemic sarcoidosis (positive histology and/or least two indirect indicators from gallium scan, chest imaging and serum angiotensin-converting enzyme); the disease is considered possible when previous criteria are not satisfied [57].

Lesions appear hyperintense on T2-weighted MRI images, located in the superficial and deep white matter; sometimes, these imaging features may resemble lesions of multiple sclerosis [58]. Also grey matter lesions are generally hyperintense on T2-weighted sequences. In the series reported by Pawate et al., contrast-enhancing parenchymal white matter lesions were found in 10 out of 54 cases (19 %) [57].

Dural thickening is generally isointense to the grey matter on T1-weighted acquisitions, hypointense on T2-weighted sequences, with contrast enhancement after gadolinium injection [59].

**Fig. 14:**
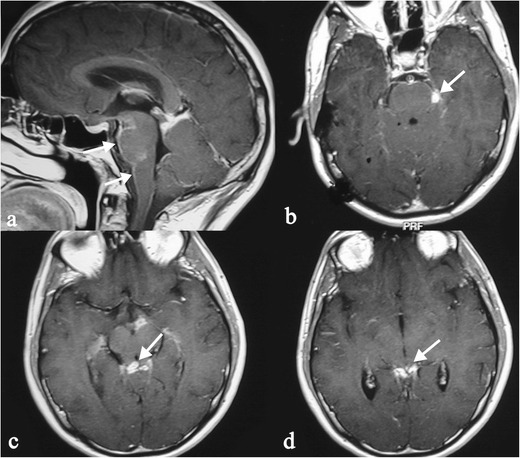
Leptomeningeal localizations of sarcoidosis. Lesions are more visible on enhanced T1-weighted acquisitions, with increased enhancement along the surface of the brainstem (*white arrows* in Fig. 14a–b). Sarcoid nodules are also visible in the superior cerebellar cistern (*white arrows* in Fig. 14c–d)

Leptomeningeal localizations are more visible after contrast injection, showing increased enhancement (Fig. 14) [58]. As reported by Urih et al., and subsequently by Christoforidis et al. [59], leptomeningeal infiltration involves frequently the suprasellar and frontal basal meninges.

Nodules can be solitary or multiple, showing enhancement after contrast injection with a ring-like appearance in the activity phase: this appearance can simulate glioblastoma or metastases. An intracranial mass presentation has been also reported in the literature, simulating a brain neoplasm [57].

Spinal sarcoidosis is reported in about 4–28 % of cases; it occurs predominantly in old people and has a poor prognosis [60]. The disease may be intramedullary or extramedullary. Junger et al. distinguish four stages of disease: 1) leptomeningeal enhancement; 2) fusiform spinal cord enlargement; 3) focal or diffuse intramedullary disease; 4) spinal cord atrophy [61].

**Fig. 15:**
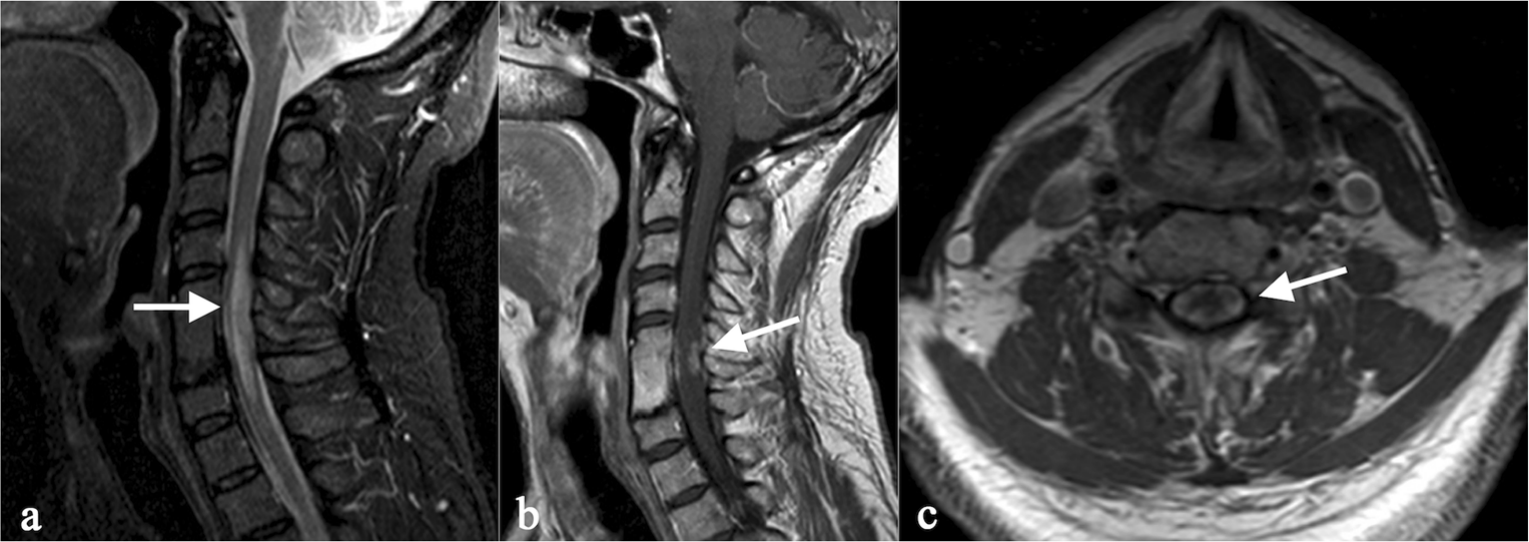
MRI images show spinal cord involvement in a 54-year-old man. T2-weighted image (Fig. 15a) demonstrates an extensive area of high signal intensity in the spinal cord (*white arrow*). On enhanced T1-weighted acquisitions, a peripheral enhancement (*white arrows*) can be depicted (Fig. 15b and c), suggesting meningeal involvement

MRI features of stage 1 and 2 disease include leptomeningeal thickening, with hyperintense signal on enhanced T1-weigheted images [60]. In cervical and thoracic regions, intramedullary lesions appear hypointense in T2-weighted MRI sequences with hyperintensity of the associated oedema; they show enhancement (Fig. 15) on T1-weighted images after contrast injection [58].

## Skeletal sarcoidosis

The skeletal system represents a rare localization of sarcoidosis [62]. Radiological features of skeletal locations have been described for the first time in 1910 by Rieder [62, 63]. It occurs in a variable range of percentages, from 9 % up to 39 % [64]. However, its frequency can be underestimated, because bone lesions are often asymptomatic [6]; frequently, the disease is misdiagnosed, being interpreted as metastatic lesions [65].

**Fig. 16:**
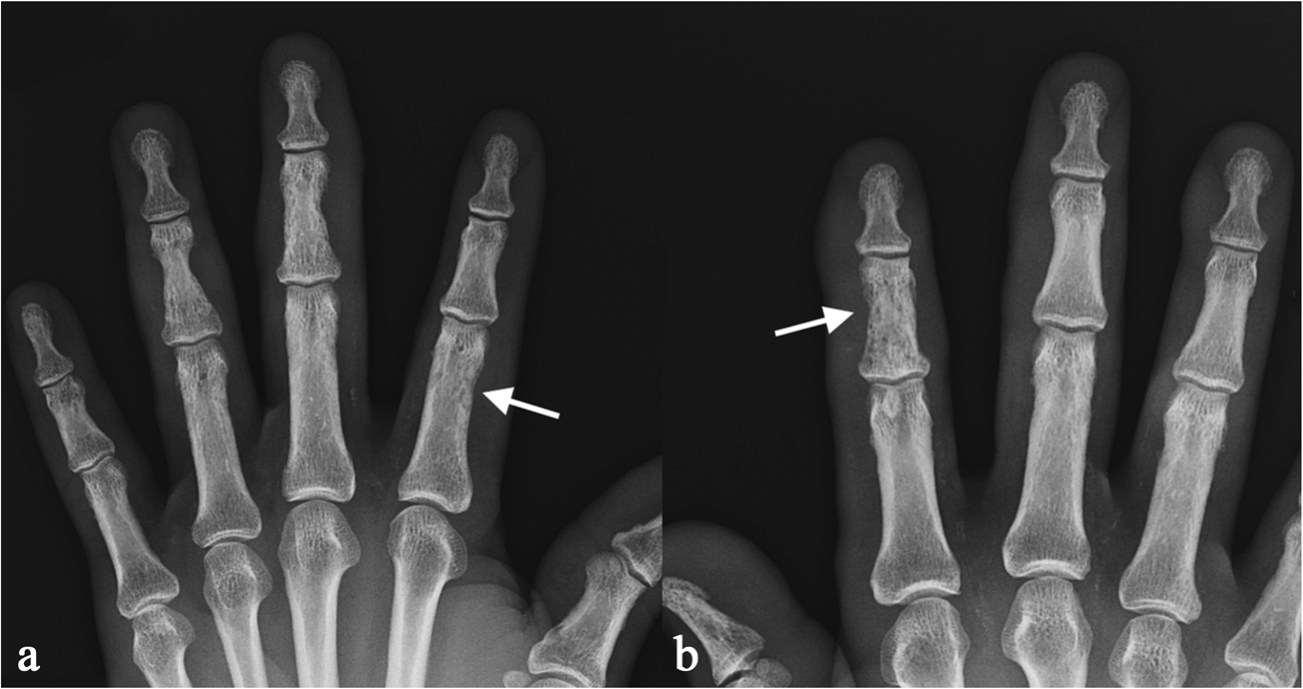
53 year-old man with hand involvement. A lacy pattern with small osteolytic areas is recognizable in the digits (*white arrows* in Fig. 16a and b)

The disease generally involves hands and feet, which have been reported in several reviews as the most affected locations [62, 64]. On conventional radiography, lesions produce a lacy pattern of osteolytic areas in the digits (Fig. 16) [19, 66–68]; cortical erosions and pathological fractures may be observed [19].

Large bones and axial skeleton involvement is uncommon; however, the disease may be encountered in ribs, long bones, skull and vertebrae. Axial skeleton disease, without pulmonary lesions, is rarely reported [6].

**Fig. 17:**
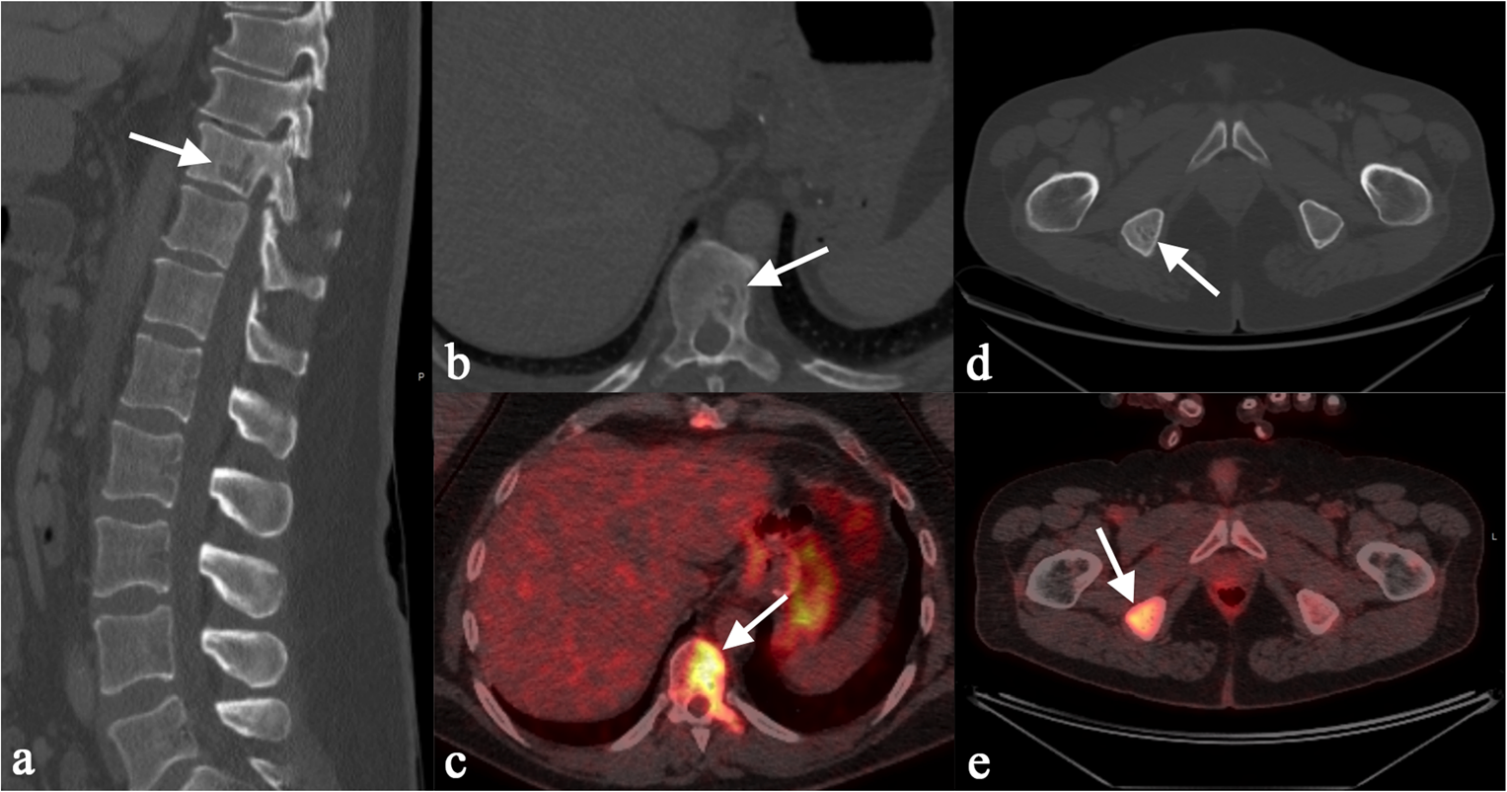
Multiple bone lesions in a 51 year-old man. Sagittal multiplanar CT image (Fig. 17a) and axial CT image (Fig. 17b) show a lytic area involving a dorsal vertebra (*white arrows*), with increased uptake on PET-CT scan (Fig. 17c). Histological examination revealed the presence of sarcoid disease of dorsal vertebra. Sarcoid lesion is also recognizable in the right ischial tuberosity (*white arrows* of Fig. 17d and e)

**Fig. 18:**
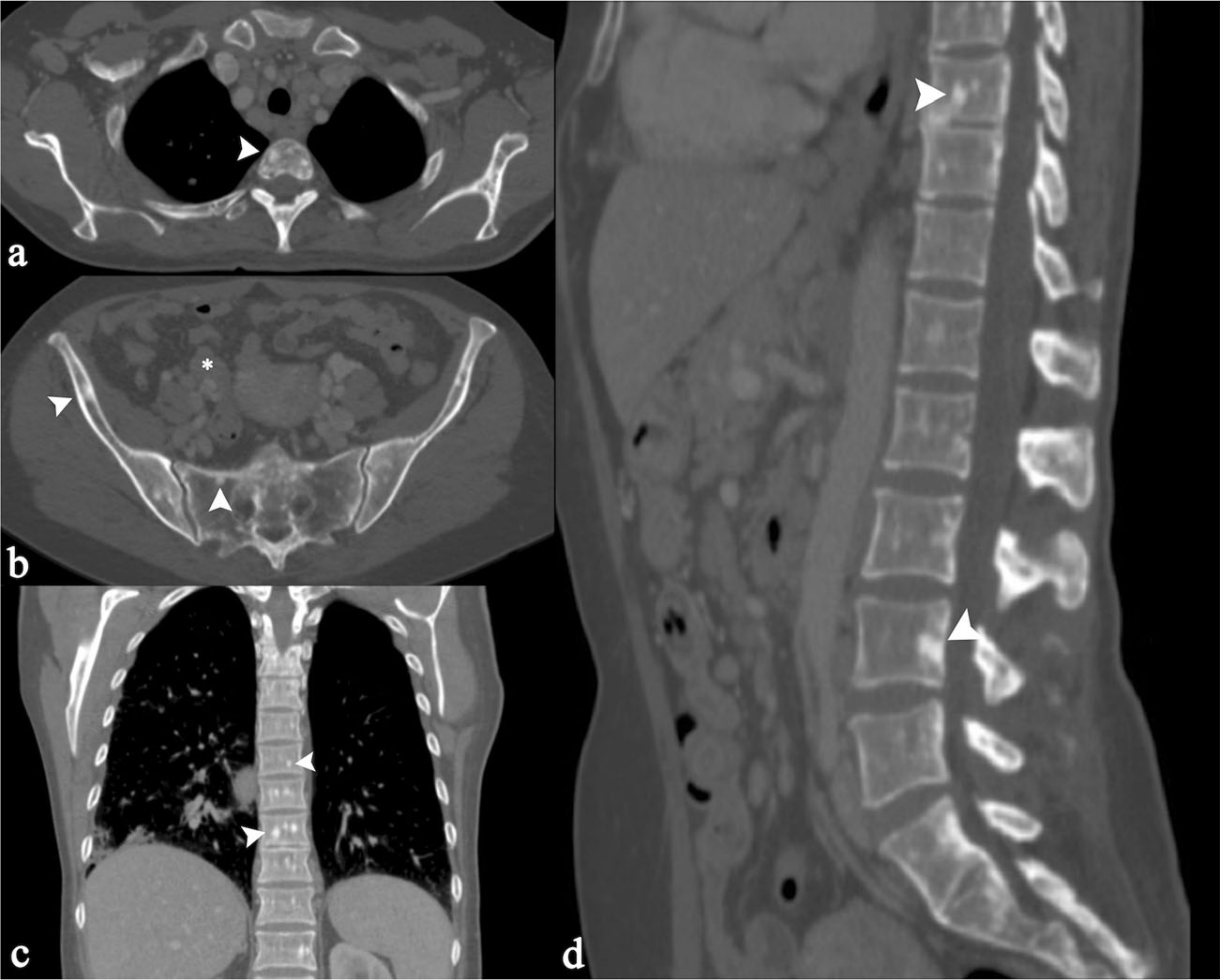
49 year-old woman with axial skeleton involvement. Axial CT images show osteosclerotic lesions (*arrowheads*) of dorsal vertebrae (Fig. 18a), sacrum (Fig. 18b), and bilateral iliac crest (Fig. 18b). A lymphadenopathy can be also observed on Fig. 18b (*white asterisk*). Coronal reconstruction clearly depicts small osteosclerotic foci in dorsal vertebrae. Sagittal reconstruction (Fig. 18d) demonstrates lesions in contiguous or non-contiguous vertebrae in the dorsal and lumbar spine

Lesions of large bone and axial skeleton can be detected as radiolucent (Fig. 17) or sclerotic areas (Fig. 18) [66]. Sclerotic areas are well depicted on CT images, consisting of hyperdense homogeneous areas, round or oval in shape (Fig. 18); osteolysis produces a hypodense appearance, and differential diagnosis from metastatic lesions is required. The MRI appearance of bone lesions is heterogeneous. Vardahanabuhuti et al. described how lesions can appear as bone marrow infiltration areas, “round, cannonball-like or intramedullary lesions”; lesions can reproduce a “starry sky appearance” [19]. Sarcoid lesions show high signal intensity on T2-weighted images, high-density proton sequences and STIR acquisitions; on T1-weighted images, lesions are generally hypointense.

**Fig. 19:**
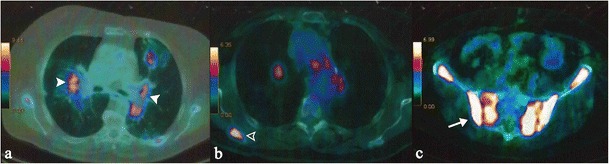
Skeletal sarcoidosis in a 71-year-old woman. Bilateral thoracic hilar lymphadenopathy is shown on PET-CT (Fig. 19a, *white arrowheads*). Increased sites of metabolic activity are also recognizable in the right scapula (Fig. 19b), in the sacrum and in the iliac crest (Fig. 19d). In presence of thoracic features of sarcoidosis, bone sites of increased uptake can be interpreted as “skeletal sarcoidosis”

In patients with sarcoidosis, skeletal FDG uptake can be observed (Fig. 19); these increased sites of metabolic activity are not specific. In combination with thoracic features of sarcoidosis, bone sites of increased uptake can be interpreted as “skeletal sarcoidosis” [36]. Muscle involvement is frequently misdiagnosed, being reported as chronic myopathy, acute myositis, or pseudotumour [69].

## Ocular sarcoidosis

Ocular involvement occurs in approximately 25–60 % of patients with systematic sarcoidosis, more frequently in the third decade (first peak of incidence) and between the sixth and the seventh decade (second peak of incidence) [70]. This may represent the initial manifestation of the disease [71], or may co-exist with asymptomatic systemic disease and can be vision-threatening.

In most cases (about 20 %), uveitis is the first common manifestation: symptoms that patients complain of include tearing, photophobia, pain, infection, lacrimation, redness. However, these symptoms can be absent: “silent uveitis” is very insidious because it may produce permanent ocular damage before treatment. The common type of uveitis is often anterior in black patients, posterior in white patients, specifically in elderly female patients [72–74]. Blindness in at least one eye occurs in about 10 % of patients and the main cause is cystoid macular oedema [75, 76].

In the anterior segment, conjunctivitis occurs in 7–70 % of the patients with ocular sarcoidosis and, together with lacrimal gland involvement, is usually asymptomatic. The sarcoidosis granulomas in the eyes have the appearance of yellow “millet-seed” nodules. When the disease involves the posterior part of the eyes, it is frequently associated with neurological manifestations: optic nerve disease, cranial nerve palsies, encephalopathy, and disorders of the hypothalamus and pituitary gland [77, 78].

**Fig. 20:**
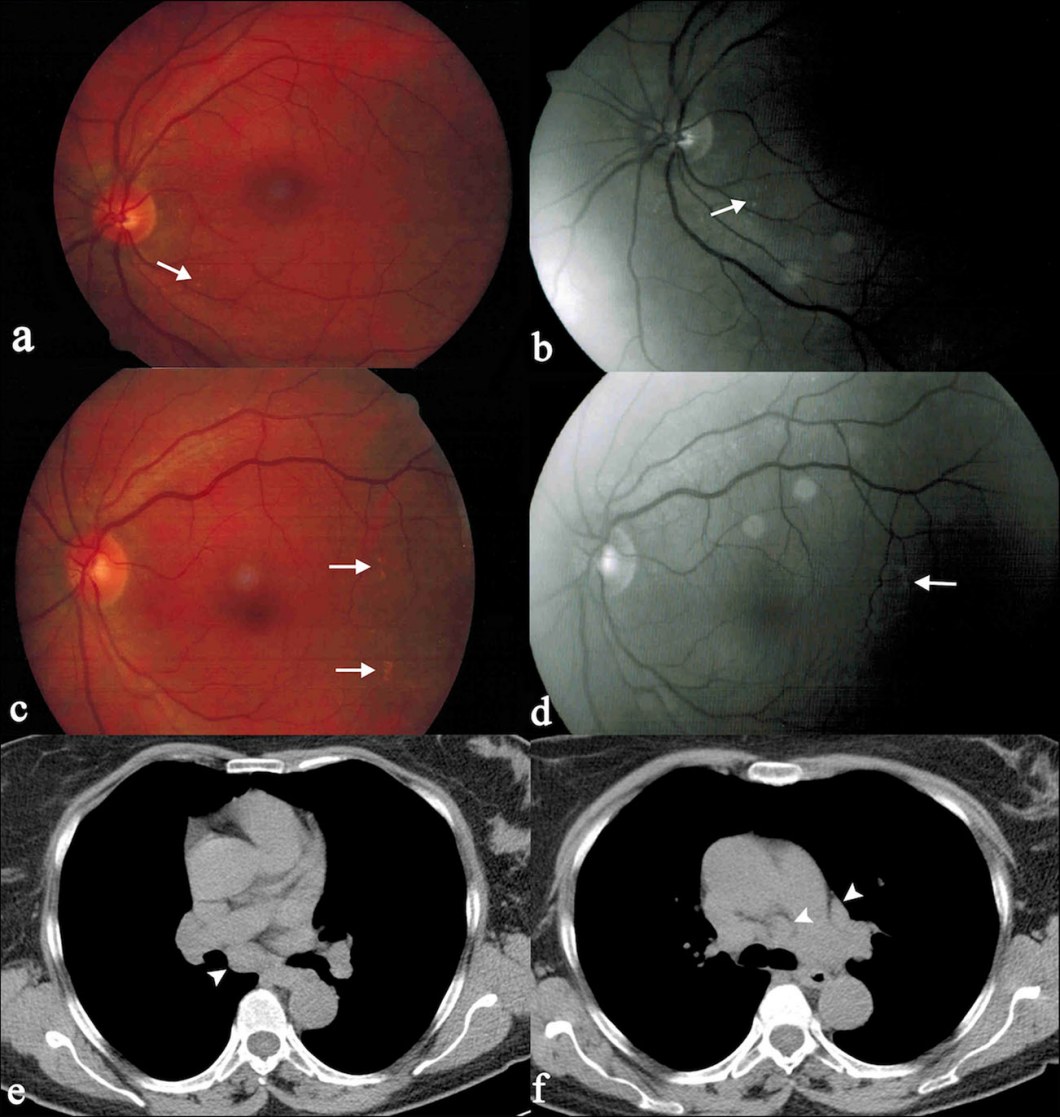
A 62-year-old woman with retinal involvement. Patient complained a left superior palpebral neoformation, which was subsequently biopsied: histological examination revealed the presence of a sarcoid nodule. She also referred to light flashes and blurred vision. Digital retinal angiography scan shows “punched-out” choroidoretinal lesions (*white arrows* on Fig. 20a–d). Chest CT (Fig. 20e and f) demonstrates mediastinal nodal enlargement, suggesting sarcoidosis in stage I

Other manifestations of ocular sarcoidosis include granulomatous iridocyclitis, retinal periphlebitis and chorioretinis; foci of retinal involvement are generally revealed by fluorangiography as “punched-out” lesions (Fig. 20). In 7 to 17 % of cases of ocular sarcoidosis occur conjunctival follicles [79, 80].

## Cutaneous sarcoidosis

In sarcoidosis, cutaneous involvement occurs in 20–35 % of patients [81]. The cutaneous lesions are distinguished as “non-specific inflammatory type” and “specific type” [82].

**Fig. 21:**
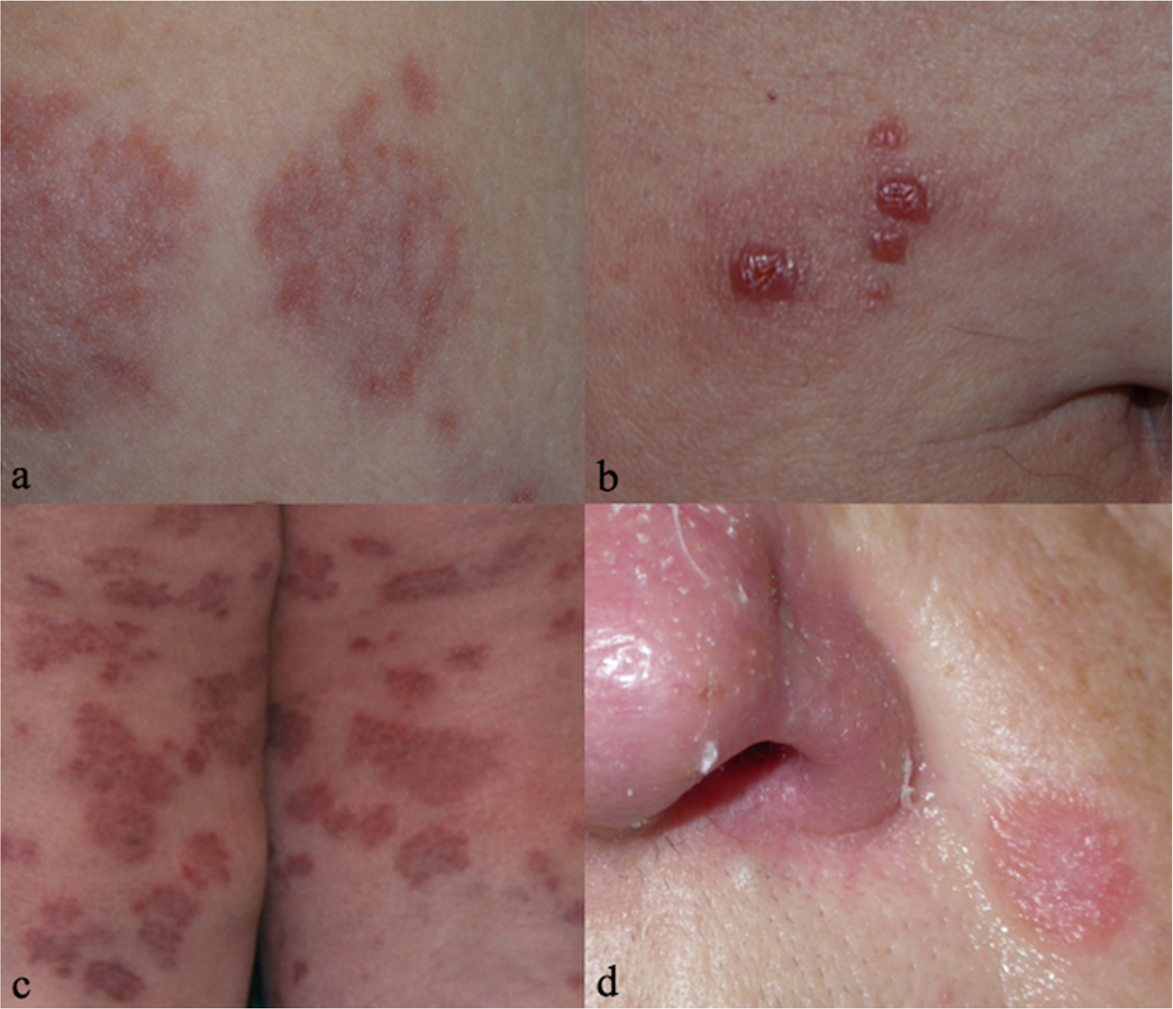
Pattern of skin involvement in different patients with sarcoidosis. Erythema nodosum occurs with subcutaneous erythematous nodules, as well recognizable on Fig. 21a; it represents the most common non-specific lesions of cutaneous disease. Papules, clearly appreciable on Fig. 21b, represent the most common “specific” cutaneous manifestation of sarcoidosis: they may occur anywhere. Multiple large annular plaques can be a cutaneous appearance of sarcoidosis (Fig. 21c). Finally, Fig. 21d shows a lupus pernio, which represents a typical clinical manifestation of cutaneous sarcoidosis

Erythema nodosum is the most common nonspecific cutaneous lesion of sarcoidosis. In most cases it occurs with subcutaneous erythematous nodules (Fig. 21a), often observed on anterior tibia, accompanied by systemic symptoms, such as fever, malaise or polyarthralgias [83].

The term “specific” is misleading, because clinically lesions are not specific to sarcoidosis, and the only way to make a diagnosis is biopsy. Papule (Fig. 21b) is the most common specific cutaneous lesion of sarcoidosis: although the face is the main location, it may occur anywhere and may be of various colours. Larger and flat-topped plaques (Fig. 21c) are very frequent and can be single or multiple; the lesions are located on the face, trunk or extremities.

A characteristic clinical manifestation of cutaneous sarcoidosis is lupus pernio (Fig. 21d): it appears as chronic and indurated papules or plaques in the middle-face, particularly the alar rim of the nose.

The diagnosis of cutaneous sarcoidosis is performed with histological examination of a cutaneous biopsy, which shows the presence of sarcoidal granuloma without any cornoid lamella.

## Cardiac sarcoidosis

Cardiac involvement with clinical manifestations occurs in 2–7 % of patients, but the occult form is much more present [84–86].

Sarcoidosis characteristic granulomatous lesion manifests in any part of the heart, but the myocardium is most frequently involved [87]. In order of frequency, the areas most affected are the left ventricular wall, interventricular septum, papillary muscles, right ventricula and atria [88, 89]. Valvular involvement is rare, but the impaired functioning of the cardiac valves may occur after sarcoid reaction of the papillary muscles [88, 90]. Pericardium involvement may be present, rarely determining constrictive pericarditis [91].

**Fig. 22:**
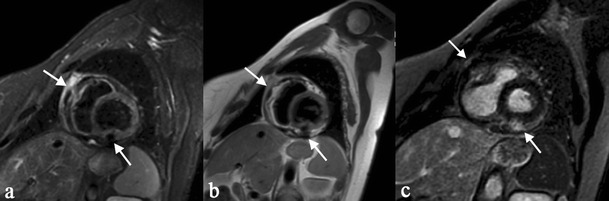
Cardiac MRI involvement in a patient with systemic sarcoidosis. Triple inversion recovery (Fig. 22a) and double-inversion recovery sequences (Fig. 22b), both obtained on short axis views, clearly show hypointense calcified myocardial and pericardial nodules (*white arrows*). After gadolinium administration, foci of delayed enhancement (*white arrows*), involving also the pericardium, are recognizable (Fig. 22c)

Gadolinium-enhanced cardiac MRI allows us to detect differences between normal tissue and altered tissue, the latter displaying areas of focal enhancement, particularly in the myocardial wall or subepicardial region (Fig. 22) [92, 93]. In addition, other MRI findings include mural oedema, pericardial effusion, myocardial or pericardial thickening and segmental wall motion abnormalities.

Radionuclide scans with gallium, citrate, and thallium are used for diagnosis and follow-up in patients with cardiac sarcoidosis [94, 95]. PET-CT is a technique for diagnosis of active involvement of cardiac sarcoidosis and can be used for the thoracic and extra-thoracic stage of the disease [96].

## Conclusions

Abdominal, neural, musculo-skeletal, cutaneous, ocular and cardiac sarcoidosis has extremely variable clinical features; their imaging findings can often simulate other diseases, namely malignancies. Sarcoidosis should be considered in the differential diagnosis of multiple lesions of brain, abdomen and bones in patients with suspected or proved disease.

In patients with pulmonary sarcoidosis, a careful evaluation of patients’ symptoms is mandatory in order to identify other locations of the disease. Radiologists, correlating imaging and clinical-pathologic findings, play an important role in diagnosis and follow-up of the disease, in order to reduce its morbidity and mortality.
